# Multiplexed single-cell 3D spatial gene expression analysis in plant tissue using PHYTOMap

**DOI:** 10.1038/s41477-023-01439-4

**Published:** 2023-06-12

**Authors:** Tatsuya Nobori, Marina Oliva, Ryan Lister, Joseph R. Ecker

**Affiliations:** 1 Plant Biology Laboratory, The Salk Institute for Biological Studies, La Jolla, CA USA; 2 Genomic Analysis Laboratory, The Salk Institute for Biological Studies, La Jolla, CA USA; 3 ARC Centre of Excellence in Plant Energy Biology, School of Molecular Sciences, University of Western Australia, Perth, Western Australia Australia; 4 The Harry Perkins Institute of Medical Research, QEII Medical Centre and Centre for Medical Research, The University of Western Australia, Perth, Western Australia Australia; 5 Howard Hughes Medical Institute, The Salk Institute for Biological Studies, La Jolla, CA USA

**Keywords:** Fluorescence in situ hybridization, Plant molecular biology, Gene expression profiling, Plant development, Plant stress responses

## Abstract

Retrieving the complex responses of individual cells in the native three-dimensional tissue context is crucial for a complete understanding of tissue functions. Here, we present PHYTOMap (plant hybridization-based targeted observation of gene expression map), a multiplexed fluorescence in situ hybridization method that enables single-cell and spatial analysis of gene expression in whole-mount plant tissue in a transgene-free manner and at low cost. We applied PHYTOMap to simultaneously analyse 28 cell-type marker genes in *Arabidopsis* roots and successfully identified major cell types, demonstrating that our method can substantially accelerate the spatial mapping of marker genes defined in single-cell RNA-sequencing datasets in complex plant tissue.

## Abstract (web summary)

This study introduces a new multiplexed fluorescence in situ hybridization method, PHYTOMap, that enables single-cell and spatial analysis of gene expression in whole-mount plant tissue in a transgene-free manner and at low cost.

## Main

Understanding how individual cells respond and interact with each other in the face of changing environments is the cornerstone of understanding tissue function. Single-cell transcriptomics technologies have been widely adopted in plant research, enabling the classification of cells into populations that share molecular features for the in-depth analysis of cell types and states^1–3^. Increasing throughput and sensitivity in single-cell transcriptomics technologies will offer tremendous granularity at which cells can be classified, but will also create new challenges in dealing with cell populations that our current histological and physiological understanding of plant cells cannot account for. To understand the identity and function of molecularly defined cell populations, it is critical to analyse their spatial localization in the tissue.

In plant research, the most common tool for spatially mapping cell population marker genes identified in single-cell transcriptome analysis has been transgenic reporter lines that express fluorescent proteins under the predicted promoter region of the genes. In most cases, each transgenic line visualizes the expression of only one gene. This approach has several limitations when analysing cells in complex tissue: (1) a cell type/state is not always defined by the expression of a single gene, but by the combination of many genes; (2) spatial mapping of a single gene or a few genes has difficulties in analysing multiple cell types/states simultaneously, which is critical for understanding interactions between cell types/states; (3) generation of transgenic plants is time-consuming; and (4) heterologous expression of fluorescent proteins does not necessarily reflect the true expression of the gene because the reporter cassettes lack the native genomic context (for example, enhancer–promoter interactions). In situ hybridization, another popular approach in spatial gene expression analysis in plants^4^, can overcome a few of the above limitations but suffers from low multiplexing capacity. Therefore, spatial gene expression analysis needs to be done with a large number of genes at single-cell resolution for a more complete understanding of the function of cell types/states and their interactions with other cells and the environment.

Spatial transcriptomics technologies hold great promise in addressing these problems by simultaneously revealing the molecular details and spatial location of cells in complex tissues. Methods using spatially barcoded arrays or imaging-based, highly multiplexed single-molecule fluorescence in situ hybridization allow researchers to study the expression of many genes (from dozens to the whole transcriptome) with spatial information (from tissue region to single-cell levels)^5^. Such technologies have recently been adopted in plant research^6–8^. Although spatial transcriptomics, combined with single-cell transcriptomics, will contribute to elucidating the spatial organization of cell types/states in plants in great detail^9^, tissue types amenable for spatial transcriptomics experiments are limited to thin (single-cell layer) sections, posing challenges for its application in plants and other organisms. For instance, the root tip—an important organ for plant growth, nutrient acquisition and interactions with microbes—is a difficult tissue to section owing to its small size. Moreover, sectioning will lead to a loss of information from other parts of the tissue, which may contain the cell types/states of interest; information about environments, such as microbial colonization, can also be lost by sectioning. It may be possible to overcome these problems by sampling serial sections and conducting multiple experiments followed by the three-dimensional (3D) reconstitution of two-dimensional (2D) data, but spatial transcriptomics technologies are very costly, making such an approach rarely affordable. To overcome these limitations, we introduce PHYTOMap (plant hybridization-based targeted observation of gene expression map), a low-cost single-cell spatial gene expression analysis that can simultaneously map dozens of genes in whole-mount plant tissue.

**Fig. 1:**
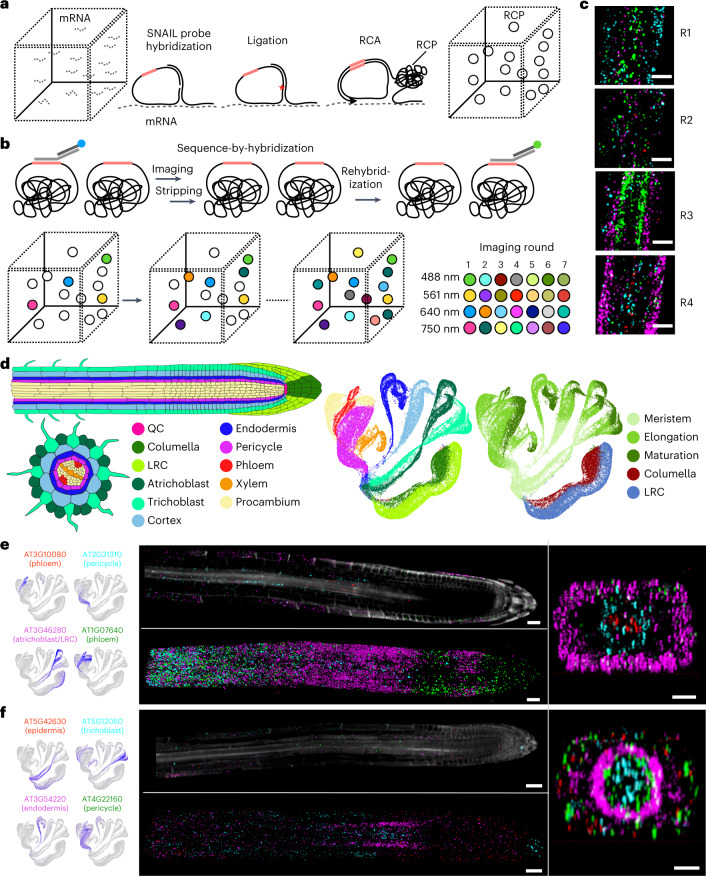
Whole-mount spatial mapping of root tip cell-type marker genes with PHYTOMap. **a**, In fixed whole-mount tissue, target mRNA molecules are hybridized by pairs of DNA probes (SNAIL probes) that harbour mRNA species-specific barcode sequences (pink bars). Barcode-containing DNA probes are circularized by ligation (red star) and amplified in situ by RCA. During amplification, amine-modified nucleotides are incorporated into the DNA amplicons (RCPs) and stably cross-linked with the cellular protein matrix using a non-reversible amine cross-linker. Amplified DNA barcodes are detected by SBH chemistry through multiple rounds of imaging. **b**, SBH chemistry. Before each imaging round, four types of bridge probes are hybridized to a set of four DNA barcodes. Each bridge probe is then targeted by one of four fluorescent probes to be imaged. After imaging, bridge probes and fluorescent probes are stripped away, keeping RCPs in place. These steps are repeated until all the DNA barcodes are read. **c**, Representative images at different imaging rounds. The maximum exposure of 60 *z* planes of the same position in the tissue is displayed. Scale bar, 30 μm. **d**, Schematic representation of the root tip and UMAPs displaying root tip scRNA-seq data^18^ used in this study. In the UMAPs, cells are labelled with cell types (left) and regions (right). LRC, lateral root cap; QC, quiescent centre. **e**,**f**, Representative results from the imaging rounds 2 (**e**) and 3 (**f**). Left, UMAPs showing expression patterns of target genes. The colours of the gene name labels correspond to the colours in the images below. Middle, 3D projections (upper) and optical sections (2D, lower) of whole-mount tissue images. Right, representative cross-section views of the middle part of the samples (transition/elongation zone). Scale bar, 25 μm.

**Extended Data Fig. 1:**
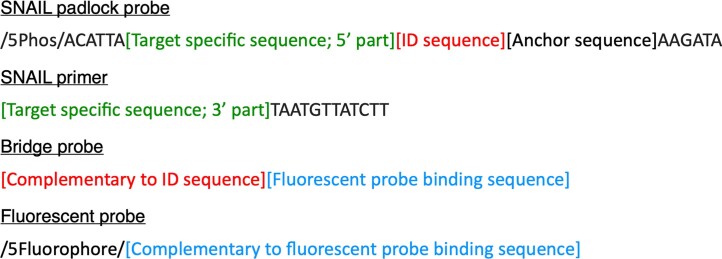
PHYTOMap probe design. ID sequences are unique to different RNA species. Anchor sequence was included based on a previous study^13^ but not used in the present study.

**Extended Data Fig. 2:**
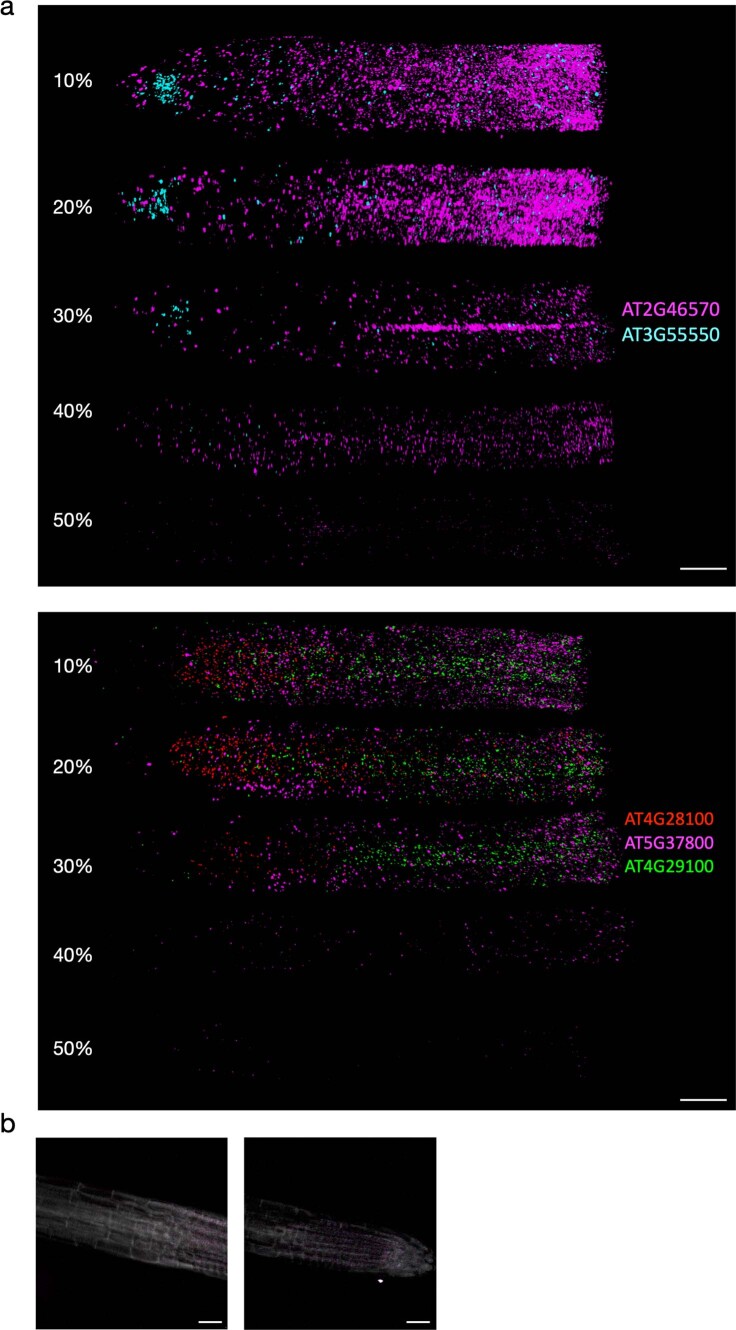
Optimization of formamide concentration during SNAIL probe hybridization. **a**, Hybridization in 30% formamide showed higher target specificity. **b**, Images after stripping fluorescent probes. The four-color channels are shown in higher contrast than in Fig. 2b, and cell wall staining images are overlaid. Scale bars = 25 μm. Three independent roots were tested with similar results.

PHYTOMap builds on in situ hybridization techniques in plants^10,11^ and in situ sequencing technologies primarily developed in neuroscience^12^. After fixing whole-mount plant tissues, DNA probes (specific amplification of nucleic acids via intramolecular ligation probes or SNAIL probes) with gene-specific barcodes are specifically hybridized on target messenger RNA molecules, circularized and amplified in situ (Fig. 1a and Extended Data Fig. 1; see Methods for details). The hybridization condition has been optimized to allow high target specificity (Extended Data Fig. 2a). The amplification of DNA barcodes provides a high signal-to-noise ratio, enabling signal detection from cleared whole-mount tissue. The location of mRNA molecules is defined using sequence-by-hybridization (SBH) chemistry^13^ that targets the barcode sequences of DNA amplicons across sequential rounds of probing, imaging and stripping (Fig. 1b). In each imaging round, four targets are detected using each of the four channels of a confocal microscope (Supplementary Video 1). After imaging, fluorescent detection probes are stripped (Extended Data Fig. 2b), and the next round of hybridization targets a new set of four genes (Fig. 1b,c). A previous study that used SBH chemistry to detect amplified DNA probes in situ showed that signal was maintained at least over 10 cycles^13^.

**Extended Data Fig. 3:**
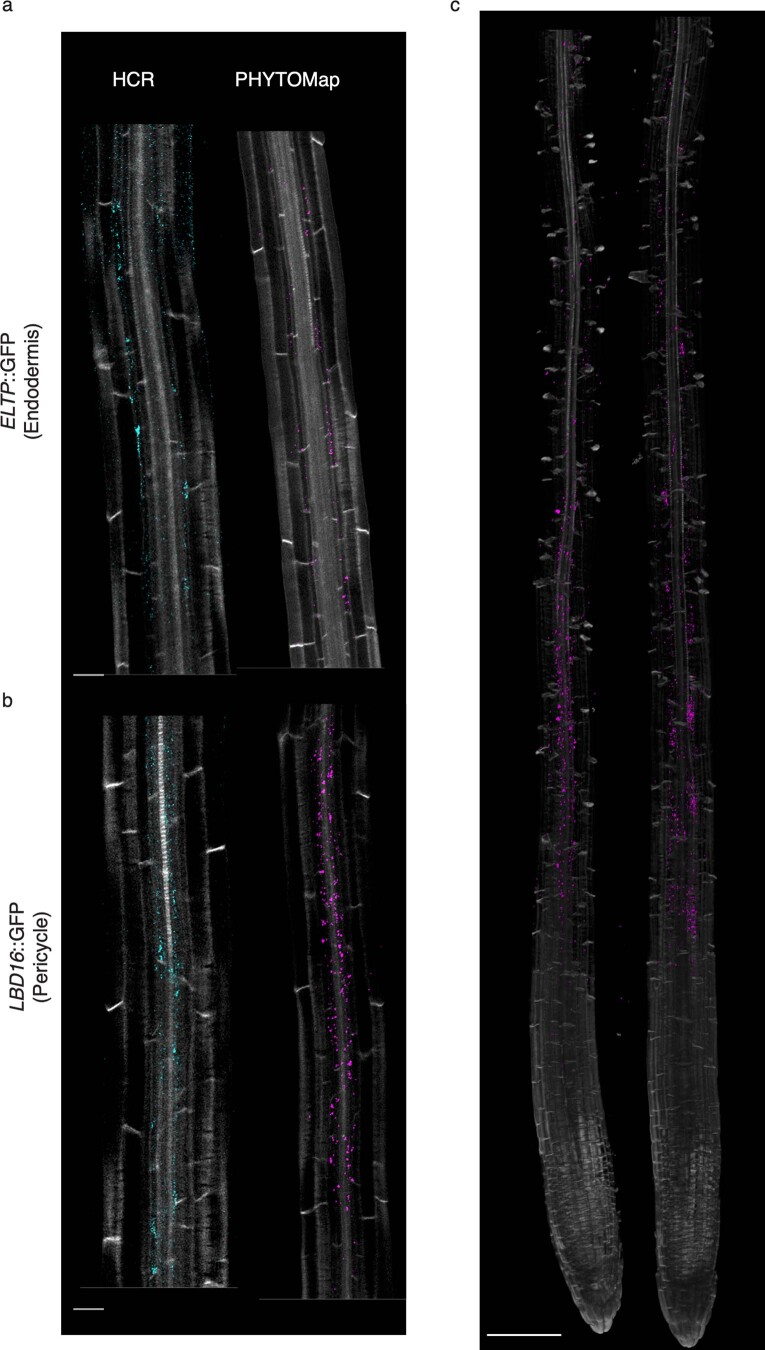
PHYTOMap validation. The mRNA of GFP was targeted with HCR (left) or PHYTOMap (right) in (**a**) *ELTP:FLS2-GFP* and (**b**) *LBD16::FLS2-GFP* plants, which express GFP in endodermis and pericycle, respectively. Scale bars = 25 μm. c, PHYTOMap images that cover larger areas. (Left) *ELTP:FLS2-GFP*. (Right) *LBD16::FLS2-GFP*. Scale bar = 100 μm. Three independent roots were tested with similar results.

We tested the accuracy of PHYTOMap by comparing its signal with results from other imaging-based techniques. We used transgenic *Arabidopsis* lines expressing green fluorescent protein (GFP) under the control of an endodermis-specific (*EMBRYO LIPID TRANSFER PROTEIN* or *ELTP*) or pericycle-specific (*LATERAL ORGAN BOUNDARIES-DOMAIN 16* or *LBD16*) promoter. Cell type-specific GFP expression in these lines has been confirmed in a previous study^14^. We targeted the mRNA of GFP with PHYTOMap and a hybridization chain reaction (HCR), which is also a hybridization-based approach recently applied to plant tissue^15^. PHYTOMap detected GFP mRNA in the expected cell types, which was further validated with HCR (Extended Data Fig. 3a,b). Together, these results confirmed the accuracy of PHYTOMap.

**Extended Data Fig. 4:**
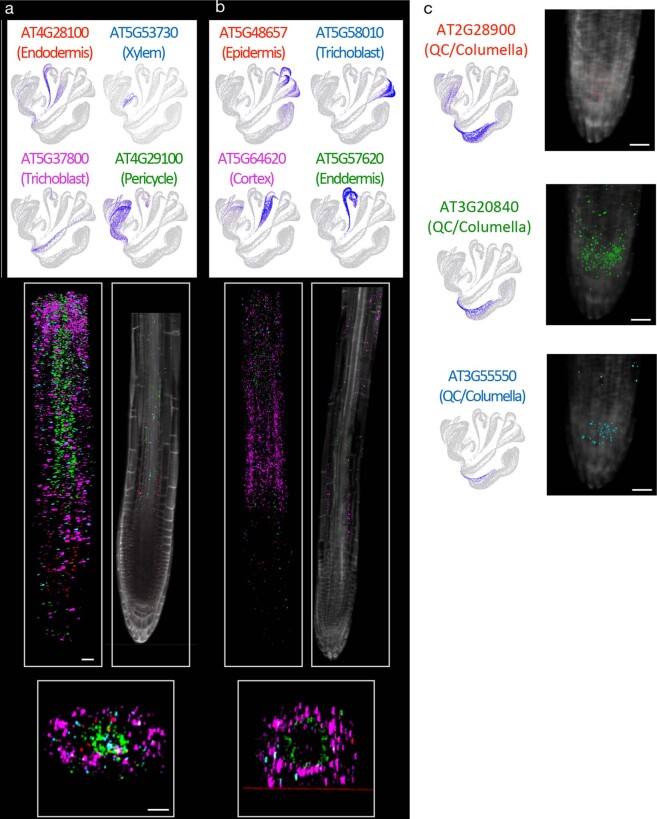
Whole-mount spatial mapping of root tip cell type marker genes predicted in scRNA-seq data with PHYTOMap. **a**, **b**, Representative results from each imaging round. Left: UMAPs showing expression patterns of target genes. The colors of gene name labels correspond to the colors in the images below. Middle: 3D projections (top) and optical sections (2D, bottom) of whole-mount tissue images. Right: Representative cross-section views of the middle part of the samples (transition/elongation zone). **c**, Validated and predicted marker genes for QC and columella. 3D images were shown with cell wall staining. Scale bars = 25 μm.

**Extended Data Fig. 5:**
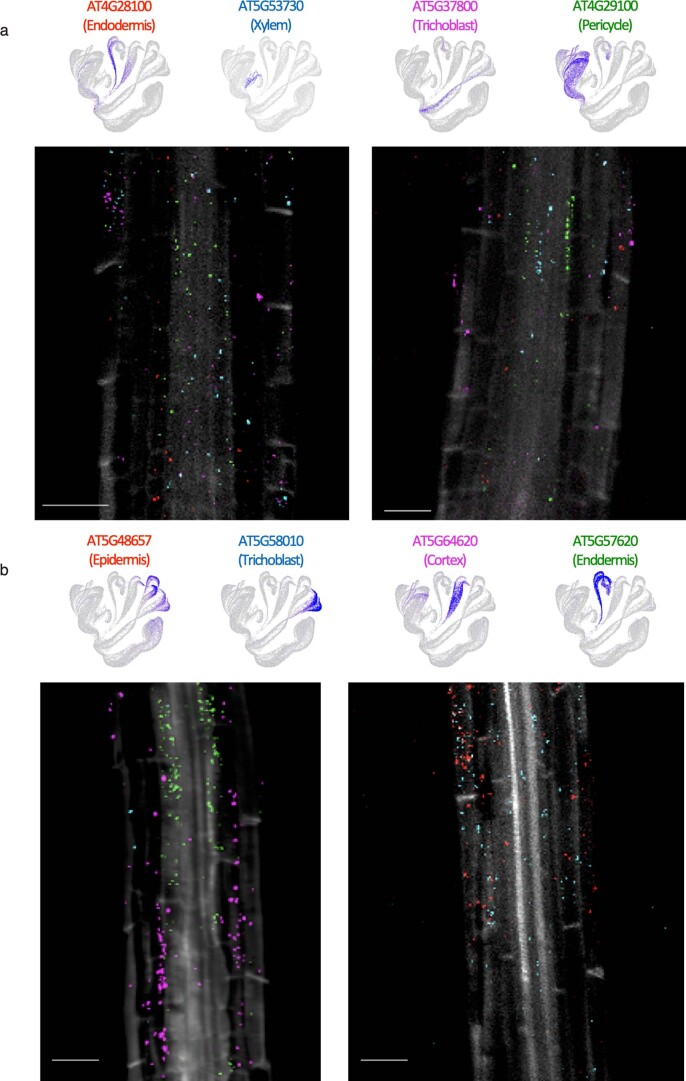
Detailed analysis of PHYTOMap images. Magnified images of 2D optical section images in (**a**) Fig. 2b and (**b**) Fig. 2. UMAP plots show expression patterns of target genes. The colors of gene name labels correspond to the colors in the images below. Scale bars = 25 μm.

**Extended Data Fig. 6:**
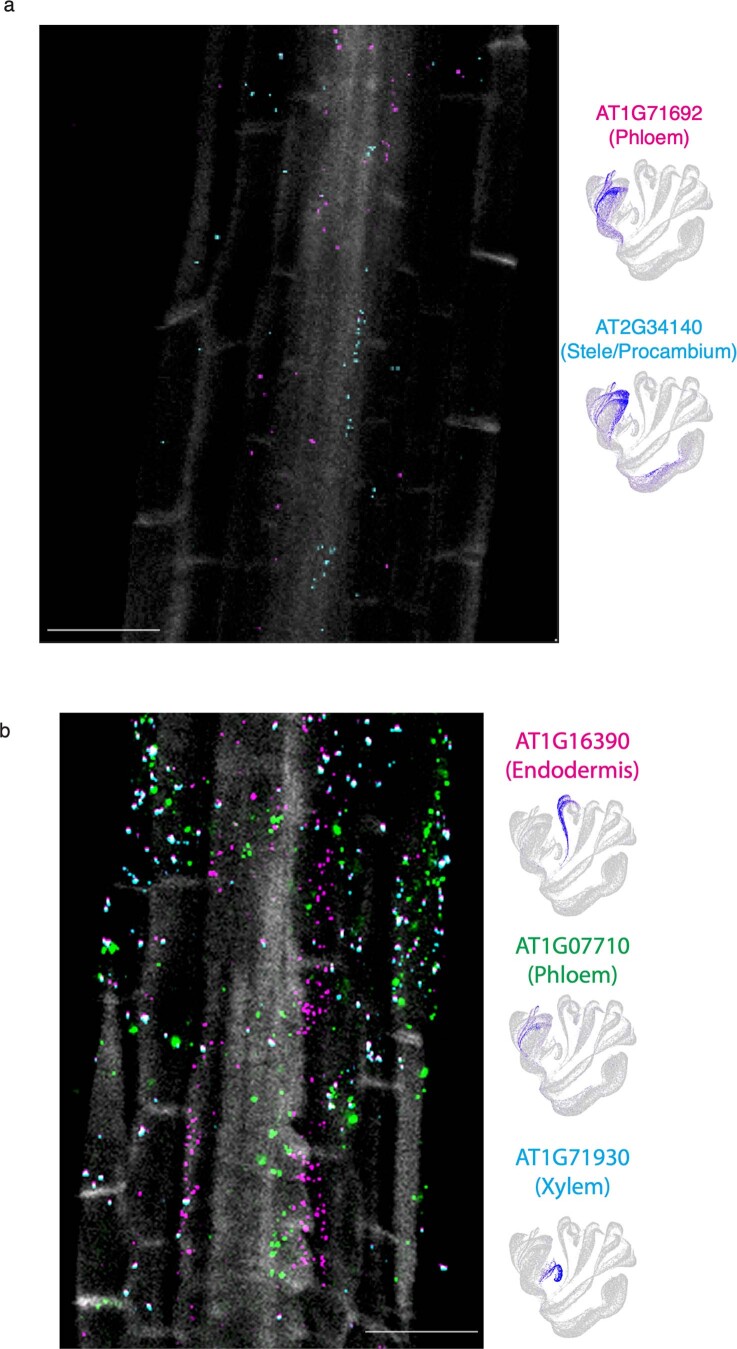
Detailed analysis of PHYTOMap images. Magnified images of 2D optical section images in (**a**) Fig. 2d, and (**b**) Fig. 2e. UMAP plots show expression patterns of target genes. The colors of gene name labels correspond to the colors in the images below. Scale bars = 25 μm.

**Extended Data Fig. 7:**
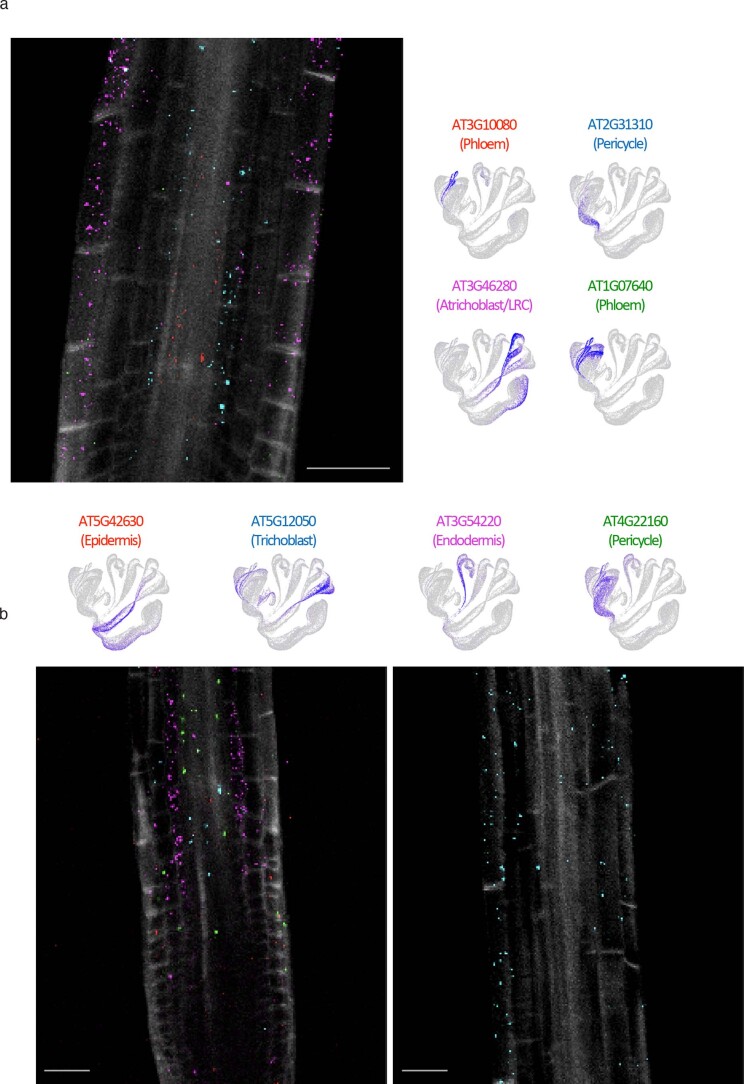
PHYTOMap images for additional genes. Magnified 2D optical section images of genes that are not shown in Fig. 1. UMAP plots show expression patterns of target genes. The colors of gene name labels correspond to the colors in the images below. Scale bars = 25 μm.

**Extended Data Fig. 8:**
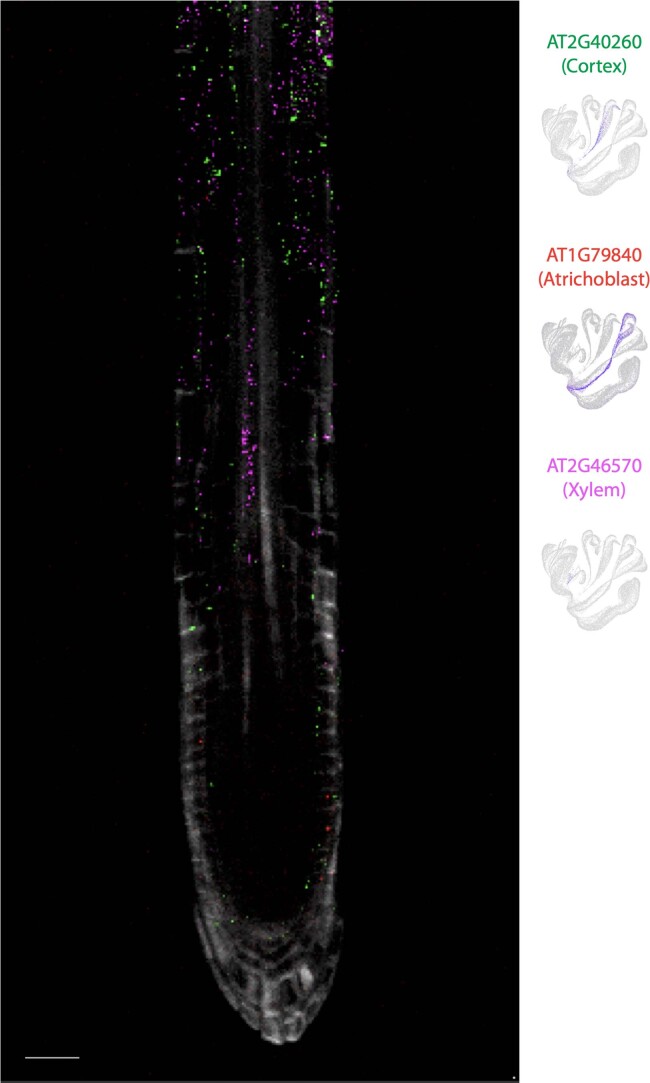
PHYTOMap images for additional genes. Magnified 2D optical section image of genes that are not shown in Fig. 1. UMAP plots show expression patterns of target genes. The colors of gene name labels correspond to the colors in the images below. Scale bars = 25 μm.

PHYTOMap successfully mapped well-established/validated cell-type marker genes in expected cell types/regions in the root tip of *Arabidopsis* (Fig. 1d–f and Extended Data Fig. 4). The marker genes we targeted include AT4G28100 (ENDODERMIS7 or EN7; endodermis), AT4G29100 (BASIC HELIX LOOP HELIX 68 or BHLH68; pericycle), AT5G37800 (RHD SIX-LIKE 1 or RSL1; trichoblast), AT5G53730 (NDR1/HIN1-LIKE 26 or NHL26; xylem), AT5G57620 (MYB DOMAIN PROTEIN 36 or MYB36; endodermis), AT5G58010 (LJRHL1-LIKE 3 or LRL3; trichoblast) and AT3G54220 (SCARECROW or SCR; endodermis) (Fig. 1e,f and Extended Data Fig. 4; magnified images are provided in Extended Data Figs. 5 and 6)^16,17^. PHYTOMap also validated cell type/region marker candidates predicted in a previous single-cell RNA-sequencing (scRNA-seq) study of *Arabidopsis* root tips^18^. For instance, AT3G46280 was detected in the root cap and elongating epidermis as predicted in the scRNA-seq data (Fig. 1e). Genes enriched in meristematic (AT5G42630) and elongation (AT5G12050) zones in the scRNA-seq data were mapped in the expected regions (Fig. 1f); AT5G12050 signal was detected in epidermis and vasculature, as shown in scRNA-seq (Fig. 1f). Quiescent center (QC) and columella signal was also detected from the marker genes AT2G28900, AT3G20840 and AT3G55550 (Extended Data Fig. 4c). Other genes that are not shown in Fig. 1 are shown in Extended Data Figs. 7 and 8. Taken together, PHYTOMap can be used as an efficient tool for validating marker genes identified in scRNA-seq data without generating transgenic plants.

**Fig. 2:**
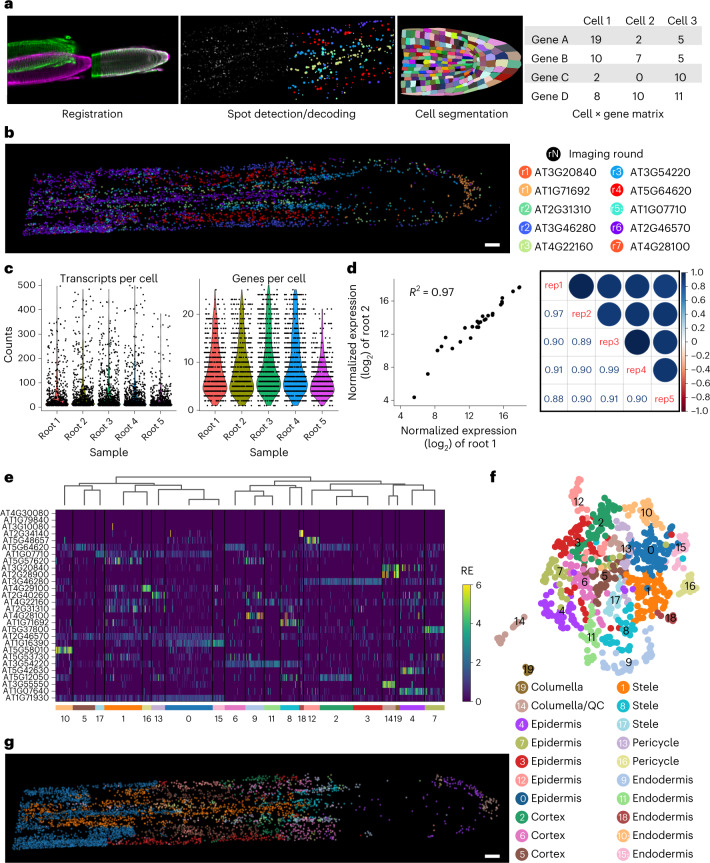
Single-cell and spatial analysis of 28 genes with PHYTOMap. **a**, PHYTOMap data analysis pipeline for single-cell analysis. **b**, 3D visualization of transcripts detected and decoded after image registration in a representative root tip (root 4). A middle section (*z* planes 90–120 of 208) of the image is displayed. Representative genes from each imaging round are shown. **c**, Violin plots showing the number of unique RNA molecules (left) and genes (right) detected in five root tip samples. **d**, Left, scatter plot comparing normalized bulk expression of each gene between two samples (root 1 and root 2). Right, correlation plot showing pair-wise correlation coefficients among five replicates. **e**, Hierarchical clustering of cells of root 4 based on the relative expression of 28 genes. Cluster IDs are indicated at the bottom. RE, relative expression. **f**, UMAP visualization of the clusters shown in **e**. **g**, 3D visualization of transcripts coloured by clusters in **e** and **f** in a representative root tip (root 4). A middle section (*z* planes 90–120 of 208) of the image is displayed. Scale bar, 25 μm (**b**,**g**).

**Extended Data Fig. 9:**
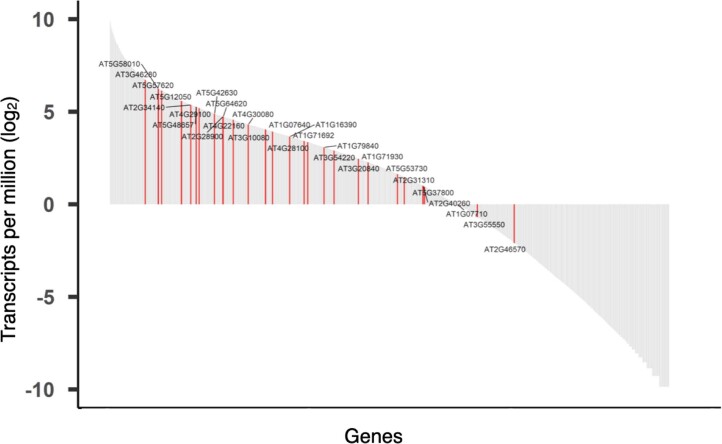
Varying levels of expression of the genes targeted in this study. Bulk expression levels of genes (transcript per million) were calculated based on the root tip scRNA-seq data^18^. The twenty-eight genes targeted in this study were highlighted in red.

To demonstrate the multiplexing capacity of this method, we simultaneously targeted 28 genes in the same root tips with seven rounds of imaging. The targeted genes include known cell-type marker genes as well as unvalidated cell-type marker candidates identified in the scRNA-seq data^18^ (a full list is given in Supplementary Table 1), which showed varying levels of expression in the root tip (Extended Data Fig. 9). We developed a computational pipeline to integrate whole-mount images from each imaging round and analyse gene expression at the single-cell resolution (Fig. 2a; see Methods for details). Cell wall boundary information was obtained together with the RNA-derived signal in each imaging round to facilitate this process. The analysis pipeline first registers 3D images across imaging rounds using cell boundary information, automatically detects spots derived from single mRNA molecules and annotates spots with gene names. A merged image with detected and decoded transcripts successfully captured the cell-type architecture of the root tip (Fig. 2b). To analyse the spatial data at the single-cell level, cell segmentation was performed based on cell wall boundary information using PlantSeg, which performs deep learning-assisted cell boundary prediction and graph partitioning-based cell segmentation^19^ (Fig. 2a). Annotated spots were assigned to individual cells and counted, resulting in a cell-by-gene matrix, a standard scRNA-seq data form that can be used for clustering and dimension reduction analyses (Fig. 2a).

We analysed five root tip preparations and identified a total of 259,781 RNA molecules from 3,608 cells (median 19 molecules per cell) (Fig. 2c). The assays were highly robust and reproducible, detecting comparable numbers of transcripts for each RNA species between different biological samples (Fig. 2d). This suggests that gene expression between cells or samples can be compared quantitatively. Hierarchical clustering and heatmap visualization revealed cell population-specific expression of target genes (Fig. 2e and Supplementary Fig. 1a). Genes that showed low expression in a previous RNA-sequencing study were detected successfully (Fig. 2e and Extended Data Fig. 9), suggesting a high sensitivity of PHYTOMap. We performed de novo clustering using PHYTOMap data and visualized the data on Uniform Manifold Approximation and Projection (UMAP) without using any spatial information (Fig. 2f and Supplementary Fig. 1b,c). These clusters successfully captured major cell types and developmental stages in the root tip (Fig. 2g). Together, these results demonstrate that PHYTOMap can spatially map dozens of genes at a single-cell resolution in a highly reproducible manner.

**Extended Data Fig. 10:**
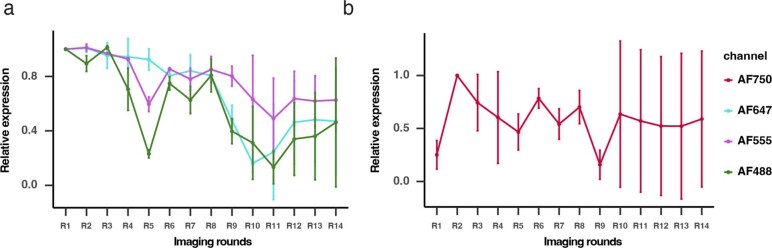
Quantitative analysis of PHYTOMap data across 14 rounds of imaging. **a**, Expression of genes labelled with Alexa Fluor 488/555/647 (Extended Data Fig. 8). Data were shown as relative expression to R1. **b**, Expression of genes labelled with Alexa Fluor 750 (Extended Data Fig. 8). Data were shown as relative expression to R2 as the data of R1 showed unusually weak signals. n = 3 independent roots. Error bars indicate standard deviation.

To test the limits of PHYTOMap, we performed 14 successive rounds of experiments targeting the same genes. We observed qualitatively consistent signals across the imaging rounds (Supplementary Fig. 2), except for one detection fluorophore (Alexa Fluor 750), whose signal decayed after the eighth round, indicating that the current protocol can detect 50 genes in the same tissue. The results also indicate that the order of imaging rounds would not substantially affect the qualitative readouts, at least in the first eight rounds. Quantitative analysis of gene expression across 14 rounds showed an overall decreasing signal and increasing noise over imaging rounds (Extended Data Fig. 10). Improving the accuracy and sensitivity of spot detection is an important future task.

In conclusion, PHYTOMap is a new technology that enables multiplexed single-cell spatial gene expression analysis in whole-mount plant tissue without requiring transgenic plant lines. A PHYTOMap experiment can be performed on a timescale similar to other in situ hybridization protocols in *Arabidopsis*^10,11^; sample preparation takes 4–5 days with ~10 h total bench time (Supplementary Fig. 3a). Imaging can be performed using a regular confocal microscope. Each imaging round takes 3 h for one root tip and 5 h for five root tips in the current study; thus 21 h and 35 h to finish imaging for a 28-gene experiment in one and five root tips, respectively. It is possible to image much larger tissues with longer imaging times. Signal could be detected from the maturation zone of the root (Extended Data Fig. 3c). PHYTOMap also successfully detected a housekeeping gene (POLYUBIQUITIN 10 or UBQ10) in whole-mount *Arabidopsis* leaves (Supplementary Fig. 4). We demonstrated that the current protocol can detect 50 genes in the same tissue. Previous studies have shown that more than 25 rounds of imaging are possible with DNA amplicons obtained using approaches similar to our method^20^, suggesting that PHYTOMap can potentially target more than 100 genes with optimized protocols. A recent study successfully reconstructed 3D spatial expression of the transcriptome of *Arabidopsis* flower meristems by integrating scRNA-seq data with validated spatial expression of 28 genes using novoSpaRc^21,22^. PHYTOMap, combined with such computational approaches, can generate a 3D spatial transcriptome atlas of various tissues and conditions. Discriminating highly similar transcripts is challenging with hybridization-based methods like PHYTOMap, but the computational approach described above can compensate for this limitation. The transgene-free nature of PHYTOMap makes this technology potentially applicable to any plant species. Cell-type annotation in scRNA-seq is challenging in many crop plants because their marker genes are often not conserved in other well-characterized species such as *Arabidopsis*. A potential challenge in applying PHYTOMap to other plant species is permeabilization of the tissue, which can be achieved by optimizing cell wall degradation protocols^23^. We believe that PHYTOMap will become a widely used tool for efficient cluster annotation in scRNA-seq studies of a variety of plant species. Beyond cell typing, PHYTOMap will offer unique opportunities to interrogate spatial regulation of complex cellular responses in plant tissue during stress and development with the ability to directly tap into various mutants that already exist.

## Methods

### Sample preparation

*Arabidopsis thaliana* accession Col-0 seeds (hereafter *Arabidopsis*) were sown on square plates containing Linsmaier and Skoog medium (Caisson Labs, catalogue no. LSP03) with 0.8% sucrose solidified with 1% agar (Caisson Labs, catalogue no. A038). Plates were kept vertically for 5 days in a growth chamber under an 8:16 h light/dark regime at 21 °C.

### PHYTOMap experimental procedure

#### Chemicals and enzymes

The following chemicals and enzymes were used: a poly-d-lysine coated dish (MatTek, catalogue no. P35GC-1.5-14-C); T4 DNA ligase (Thermo Fisher Scientific, catalogue no. EL0011); EquiPhi29 DNA polymerase (Thermo Fisher Scientific, catalogue no. A39391); SUPERaseIn RNase inhibitor (Invitrogen, catalogue no. AM2696); aminoallyl dUTP (AnaSpec, catalogue no. AS-83203); Dulbecco’s phosphate-buffered saline (DPBS) (Sigma, catalogue no. D8662); molecular biology grade BSA (New England Biolabs, catalogue no. B9000S); dNTPs (New England Biolabs, catalogue no. N0447S); Fluorescent Brightener 28 disodium salt solution (Sigma, catalogue no. 910090); formaldehyde solution for molecular biology, 36.5%–38% in water (Sigma, catalogue no. F8775); Triton-X (Sigma, catalogue no. 93443); Proteinase K (Invitrogen, catalogue no. 25530049); nuclease-free water (Invitrogen, catalogue no. AM9937); BS(PEG)9 (Thermo Fisher Scientific, catalogue no. 21582); 20× SSC buffer (Sigma-Aldrich, catalogue no. S6639); ribonucleoside vanadyl complex (New England Biolabs, catalogue no. S1402S); formamide (Sigma, catalogue no. F9037); RNase-free Tris buffer pH 8.0 (Invitrogen, catalogue no. AM9855G); RNase-free EDTA pH 8.0 (Invitrogen, catalogue no. AM9260G); cellulase (Yaklut, catalogue no. YAKL0013); macerozyme (Yakult, catalogue no. YAKL0021); and pectinase (Thermo Fisher Scientific, catalogue no. ICN19897901).

#### Probe design

Target genes were selected manually based on their cell type-specific expression. Probes were constructed by combining the probe design used in STARmap^24^ and HYBISS^13^ (Extended Data Fig. 1a). A SNAIL probe—a pair comprising a padlock probe (PLP) and a primer—was designed. (1) For each gene, 40–50-nucloetide sequences with a GC content of 40%–60% were selected and it was confirmed that there was no homologous region in the other transcripts by blasting against TAIR10 *Arabidopsis* genome. (2) Selected sequences were split into halves, each of 20–25 nucleotides (the 5′ halves for PLPs and the 3′ halves for primers), with a two-nucleotide gap between, ensuring that the melting temperature (*T*_m_) of each half is around 60 °C. (3) PLPs have complementary sequences for target specific bridge probes. (4) Four SNAIL probes were designed for each gene. (5) PLPs and primers have complementary sequences to form a circular structure. Bridge probes and detection read-out probes were designed as described previously^13^ and detailed in Supplementary Table 2. All probes were manufactured by Integrated DNA Technologies. SNAIL probes were manufactured in the form of oPools Oligo Pools with desalting purification. Bridge probes were manufactured individually with desalting purification. Detection read-out probes were manufactured individually with HPLC purification.

#### Sample fixation and permeabilization

Five-day-old root tips were cut on the agar plate using a razor blade, mounted on a dry poly-d-lysine coated dish using tweezers, and immediately fixed, dehydrated and rehydrated in a manner similar to that described in previous studies^4,15^ with modifications. The following steps were conducted on the dish. *Arabidopsis* root tips were immersed in FAA (16% v/v formaldehyde, 5% v/v acetic acid and 50% ethanol) for 1 h at room temperature. RNase-free water was used throughout the entire protocol. Samples were then dehydrated in a series of 10-min washes once in 70% (v/v in nuclease-free water) ethanol, once in 90% ethanol and twice in 100% ethanol, followed by two 10-min washes in 100% methanol, and then were stored in 100% methanol at −20 °C overnight. The next day, samples were rehydrated in a series of 5-min washes in 75% (v/v), 50% and 25% methanol in DPBS-T (0.1% Tween 20 in DPBS) at room temperature. The cell wall was partially digested by incubating samples in cell wall digestion solution (0.06% cellulase, 0.06% macerozyme, 0.1% pectinase, and 1% SUPERase in DPBS-T) for 5 min on ice, and then for 30 min at room temperature. After two washes in DPBS-TR (DPBS-T and 1% SUPERase), samples were fixed in 10% (v/v) formaldehyde for 30 min at room temperature and washed with DPBS-TR. Proteins were digested by incubating samples in protein digestion buffer (0.1 M Tris–HCl pH 8, 50 mM EDTA pH 8) with a 1:100 volume of Proteinase K (20 mg ml^−1^, RNA grade; Invitrogen, catalogue no. 25530049) for 30 min at 37 °C. After two washes in DPBS-TR, samples were fixed in 10% (v/v) formaldehyde for 30 min at room temperature and washed with DPBS-TR.

#### SNAIL probe hybridization, amplification and fixation

The following steps are based on STARmap protocols^24^ with modifications. A pool of SNAIL probes (500 nM each) was heated at 90 °C for 5 min and cooled at room temperature. Samples were incubated in hybridization buffer (2× SSC, 30% formamide, 1% Triton-X, 20 mM ribonucleoside vanadyl complex and pooled SNAIL probes at 10 nM per oligo) in a 40 °C humidified oven overnight. After hybridization, samples were washed twice in DPBS-TR and once in 4× SSC in DPBS-TR for 30 min at 37 °C and rinsed with DPBS-TR at room temperature. Samples were then incubated in a T4 DNA ligation mixture (1:50 dilution of T4 DNA ligase supplemented with 1× BSA and 0.2 U µl^−1^ of SUPERase-In) at room temperature overnight. After ligation, samples were washed twice with DPBS-TR for 10 min at room temperature and incubated in a rolling circle amplification (RCA) mixture (1:20 dilution of equiPhi29 DNA polymerase, 250 µM dNTP, 0.1 µg µl^−1^ BSA, 1 mM dithiothreitol, 0.2 U µl^−1^ of SUPERase-In and 20 µM aminoallyl dUTP) at 37 °C overnight. After RCA, samples were rinsed in DPBS-T and covalently cross-linked with 4.3 µg µl^−1^ BS(PEG)9 in DPBS-T. BS(PEG)9 was then quenched by incubating samples in 1 M Tris–HCl (pH 8) for 30 min at room temperature.

#### Gel embedding and tissue clearing

After the fixation of DNA amplicons, samples were embedded in acrylamide gel by incubating in a polymerization mixture (4% acrylamide, 0.2% bis-acrylamide, 0.1% ammonium persulfate and 0.1% tetramethylethylenediamine in DPBS-T) for 1.5 h at room temperature. Samples were then rinsed in DPBS-T. After gel embedding, samples were cleared by incubating in ClearSee^25^ at room temperature overnight.

#### Sequence-by-hybridization

Samples were washed with 2× SSC for 5 min at room temperature and then incubated in a bridge probe hybridization mixture (2× SSC, 20% formamide and four bridge probes at 100 nM per oligo in water) for 1 h at room temperature. After washing twice in 2× SSC for 5 min at room temperature, samples were incubated in a detection probe hybridization mixture (2× SSC, 20% formamide, 1:100 dilution of Calcofluor White (Fluorescent Brightener 28 disodium salt solution) and fluorescent detection oligos at 100 nM per oligos in water) for 1 h at room temperature. Samples were washed in 2× SSC and ClearSee for 5 min at room temperature and stored in ClearSee until imaging. After imaging, the PHYTOMap signal was stripped by incubating in stripping buffer (65% formamide in 2× SSC) at 30 °C for 30 min.

#### Imaging

Imaging was performed using a Leica Stellaris 8 confocal microscope equipped with a DMi8 CS Premium, supercontinuum white light laser, laser 405 DMOD, power HyD detectors and an HC PL APO CS2 ×40/1.10 water objective. The image size for a field-of-view was 512 × 512 pixels with a voxel size of 0.57 μm × 0.57 μm × 0.42 μm, and three fields-of-view were acquired for each root sample unless otherwise stated. The 2D images shown in Extended Data Fig. 4b were taken in a scan format of 2,048 × 2,048 pixels with denoising (averaging two images). The following channel settings were used: 405 nm excitation, 420–510 nm emission; 499 nm excitation, 504–554 nm emission; 554 nm excitation, 559–650 nm emission; 649 nm excitation, 657–735 nm emission; 752 nm excitation, 760–839 nm emission.

#### PHYTOMap in the leaf

*Arabidopsis* plants were grown in soil for 20 days with a 12 h light period. The fifth leaf (the largest) was used for the experiment. Leaves were processed as described above with slight modifications. Because the whole-mount leaf did not attach to the poly-d-lysine coated dish, the tissue was fixed in a 1.5 ml tube with FAA. A vacuum was applied to facilitate fixation. After the first fixation, the tissue was transferred to a poly-d-lysine coated dish and the downstream steps were carried out on the dish. The tissue was not embedded in the gel, because we did not perform multiple rounds of imaging. Before imaging, the tissue was mounted on a glass slide with a coverslip on top to immobilize the tissue. SNAIL probes for UBQ10 (AT4G05320) were used (Supplementary Table 2).

#### Cost of PHYTOMap

The cost of PHYTOMap experiments is approximately US$80 for a 28-gene experiment and US$230 for a 96-gene experiment (Supplementary Fig. 3b and detailed in Supplementary Table 3), where each experiment can accommodate five or more root tips, which can be from different treatments and/or genotypes. The initial investment (reagent cost) to set up PHYTOMap experiments is approximately US$2,700 and US$5,500 for a 28-gene and 96-gene experiment, respectively.

### PHYTOMap data processing

#### Image registration

Sample handling could cause shifts in a field-of-view during image acquisition. To correct these shifts, image stacks from each round were registered in three dimensions based on the cell wall boundary staining information by a global affine alignment using random sample consensus-based feature matching^26^. We adopted the analysis pipeline of *Bigstream*^27^ with modifications. The first round of images was used as a reference. The registered images were used for downstream analysis with *starfish* (https://github.com/spacetx/starfish), a Python library for processing image-based spatial transcriptomics data.

#### Spot detection and decoding

Registered image stacks were processed with ImageJ (v.2.3.0) into individual images for each channel and z-step that *starfish* can process. Images were denoised using the *Bandpass* function, and the z axis was smoothed by Gaussian blurring using the *GaussianLowPass* function with the following parameters: lshort = 0.5, llong = 11 and threshold=0.0. Using the *Clip* function, an image clipping filter was applied to remove pixels of too low or too high intensity. Fluorescence in situ hybridization signals (spots) from single molecule-derived rolling circle products (RCP) were detected by a blob detection technique using the *BlobDetector* function, which is a multidimensional Gaussian spot detector that convolves kernels of multiple defined sizes with images to identify spots. The kernel sizes were determined based on the diameter of spots (typically around 1 µm). Detected spots were decoded based on the imaging round and the channel information using the *SimpleLookupDecoder* function.

#### Cell segmentation

The cell wall staining image of the first imaging round (the same image used as a reference for image registration) was used for segmentation. PlantSeg workflow^19^ was used to predict cell boundaries and label the cells in the image stacks. A re-scaling factor of [1.68, 2.28, 2.28] was used to fit our images to the ‘confocal_PNAS_3d’ model on the software. A graphics processing unit-based convolutional neural network prediction was used for cell boundary prediction with the patch size of [80, 160, 160] and the ‘accurate’ mode (50% overlap between patches). The Multicut segmentation algorithm was used with under-/oversegmentation factor = 0.5, 3D watershed, convolutional neural network predictions threshold = 0.3, watershed seeds sigma = 1.0, watershed boundary sigma = 0, superpixels minimum size = 1, and cell minimum size = 1. After segmentation, images were re-scaled with the appropriate factors.

#### Spot assignment to segmented cells

Based on the segmentation masks generated in the previous step, individual decoded spots were assigned to cells using the *AssignTargets* function. The spots were then counted for each target in each cell, resulting in a cell-by-gene matrix.

#### Image visualization

Registered and decoded images were visualized using *napari*^28^, a fast, interactive, multidimensional image viewer for Python, by using the *starfish* function *display*.

### PHYTOMap count data analysis

*scanpy* was used for analysing count data^29^. Cells that contain fewer than six spots (transcripts) were filtered out from the analysis. Count data were log-transformed, and principal components were calculated. A neighbourhood graph was computed by using 10 principal components with a local neighbourhood size of five. UMAP embedding was generated based on the neighbourhood graph. Clustering was performed with the Leiden algorithm with a parameter resolution of 1. The plots in Extended Data Fig. 10 were created using ggplot2 (v.3.3.5).

### HCR

HCR was performed as reported previously^15^ with some modifications. Root tips were fixed and permeabilized as described above in the PHYTOMap method. After protein digestion and post fixation, the sample was pre-incubated in HCR probe hybridization buffer (Molecular Instruments, catalogue no. BPH02323) for 30 min at 37 °C, then incurvated in HCR probe hybridization buffer with a 1:500 volume of a GFP-targeting probe mixture (designed by Molecular Instruments) overnight at 37 °C. After probe hybridization, the sample was washed twice with HCR probe wash buffer (Molecular Instruments, catalogue BPH01923) for 30 min at 37 °C and twice with 5× SSCTR (5x SSC, 0.1% Tween and 0.2 U µl^−1^ of SUPERase-In) for 10 min at room temperature. The sample was then incubated in the HCR amplification buffer (Molecular Instruments, catalogue number BAM02323) for 30 min at room temperature. During the incubation, HCR amplifier B3-h1/2 Alexa Fluor 647 was heated to 95 °C for 90 s in a thermocycler and cooled at room temperature for 30 min. The amplification solution was prepared by adding a 1:50 volume of cooled HCR amplifiers to the HCR amplification buffer. The sample was incubated in the amplification solution overnight at room temperature and washed three times with 5× SSCTR for 20 min at room temperature. The sample was then cleared in ClearSee for more than 1 day until imaging. For imaging, the cell wall of the samples was stained with Calcofluor White as described above.

### scRNA-seq analysis

Processed and annotated data by Shahan et al.^18^ were downloaded from the Gene Expression Omnibus (GSE152766_Root_Atlas_spliced_unspliced_raw_counts.rds.gz). The R package Seurat (v.4.1.0)^30^ was used to display the expression of target genes.

### Reporting summary

Further information on research design is available in the Nature Portfolio Reporting Summary linked to this article.

## Supplementary information

Supplementary Information Supplementary Figs. 1–4.

Reporting Summary

Supplementary Video Representative 3D images of root tips visualized by PHYTOMap.

Supplementary Table Supplementary Tables 1–3.

## Data Availability

Image data are available at http://neomorph.salk.edu/downloads/phytomap/. Sequences of all the DNA probes used in this study are provided in Supplementary Table 2. Processed and annotated scRNA-seq data is available at the Gene Expression Omnibus (GSE152766).

## Code availability

The code to analyse PHYTOMap data is available at https://github.com/tnobori/PHYTOMap.

## References

[1] Cole B (2021). Plant single-cell solutions for energy and the environment.. Commun. Biol..

[2] Seyfferth C (2021). Advances and opportunities of single-cell transcriptomics for plant research.. Annu. Rev. Plant Biol..

[3] Birnbaum KD (2018). Power in numbers: single-cell RNA-seq strategies to dissect complex tissues. Annu. Rev. Genet..

[4] Rozier F, Mirabet V, Vernoux T, Das P (2014). Analysis of 3D gene expression patterns in plants using whole-mount RNA in situ hybridization. Nat. Protoc..

[5] Rao A, Barkley D, França GS, Yanai I (2021). Exploring tissue architecture using spatial transcriptomics. Nature.

[6] Giacomello S (2017). Spatially resolved transcriptome profiling in model plant species. Nat. Plants.

[7] Laureyns R (2022). An in situ sequencing approach maps PLASTOCHRON1 at the boundary between indeterminate and determinate cells. Plant Physiol..

[8] Xia, K. et al. The single-cell stereo-seq reveals region-specific cell subtypes and transcriptome profiling in *Arabidopsis* leaves. *Dev. Cell* **57**, 1299–1310 (2022). 10.1016/j.devcel.2022.04.011 35512702

[9] Cox, K. L. et al. Organizing your space: the potential for integrating spatial transcriptomics and 3D imaging data in plants. *Plant Physiol.* **188**, 703–712 (2022). 10.1093/plphys/kiab508 PMC8825300 34726737

[10] Hejátko J (2006). In situ hybridization technique for mRNA detection in whole mount *Arabidopsis* samples. Nat. Protoc..

[11] Brewer PB, Heisler MG, Hejátko J, Friml J, Benková E (2006). In situ hybridization for mRNA detection in *Arabidopsis* tissue sections. Nat. Protoc..

[12] Moses L, Pachter L (2022). Museum of spatial transcriptomics. Nat. Methods.

[13] Gyllborg D (2020). Hybridization-based in situ sequencing (HybISS) for spatially resolved transcriptomics in human and mouse brain tissue. Nucleic Acids Res..

[14] Wyrsch I, Domínguez-Ferreras A, Geldner N, Boller T (2015). Tissue-specific FLAGELLIN-SENSING 2 (FLS2) expression in roots restores immune responses in *Arabidopsis*
*fls2* mutants. New Phytol..

[15] Oliva, M. et al. An environmentally-responsive transcriptional state modulates cell identities during root development. Preprint at https://www.biorxiv.org/content/early/2022/03/04/2022.03.04.483008 (2022).

[16] Wendrich JR (2020). Vascular transcription factors guide plant epidermal responses to limiting phosphate conditions. Science.

[17] Menand B (2007). An ancient mechanism controls the development of cells with a rooting function in land plants. Science.

[18] Shahan, R. et al. A single-cell *Arabidopsis* root atlas reveals developmental trajectories in wild-type and cell identity mutants. *Dev. Cell* **57**, 543–560 (2022). 10.1016/j.devcel.2022.01.008 PMC9014886 35134336

[19] Wolny A (2020). Accurate and versatile 3D segmentation of plant tissues at cellular resolution. eLife.

[20] Lee, J. H. et al. Fluorescent in situ sequencing (FISSEQ) of RNA for gene expression profiling in intact cells and tissues. *Nat. Protoc.* **10**, 442–458 (2015). 10.1038/nprot.2014.191 PMC4327781 25675209

[21] Neumann, M. et al. A 3D gene expression atlas of the floral meristem based on spatial reconstruction of single nucleus RNA sequencing data. *Nat. Commun.* **13**, 2838 (2022). 10.1038/s41467-022-30177-y PMC9122980 35595749

[22] Nitzan M, Karaiskos N, Friedman N, Rajewsky N (2019). Gene expression cartography. Nature.

[23] Giacomello S, Lundeberg J (2018). Preparation of plant tissue to enable Spatial Transcriptomics profiling using barcoded microarrays. Nat. Protoc..

[24] Wang X (2018). Three-dimensional intact-tissue sequencing of single-cell transcriptional states. Science.

[25] Kurihara D, Mizuta Y, Sato Y, Higashiyama T (2015). ClearSee: a rapid optical clearing reagent for whole-plant fluorescence imaging. Development.

[26] Fischler MA, Bolles RC (1981). Random sample consensus. Commun. ACM.

[27] Wang Y (2021). EASI-FISH for thick tissue defines lateral hypothalamus spatio-molecular organization. Cell.

[28] Sofroniew, N. et al. napari: a multi-dimensional image viewer for Python. *Zenodo*10.5281/zenodo.6598542 (2022).

[29] Wolf, F. A., Angerer, P. & Theis, F. J. SCANPY: large-scale single-cell gene expression data analysis. *Genome Biol.* **19**, 15 (2018). 10.1186/s13059-017-1382-0 PMC5802054 29409532

[30] Butler A, Hoffman P, Smibert P, Papalexi E, Satija R (2018). Integrating single-cell transcriptomic data across different conditions, technologies, and species. Nat. Biotech..

