# Supplementary material for: Multiplexed single-cell 3D spatial gene expression analysis in plant tissue using PHYTOMap
Source: Nat Plants. 2023 Jun 12;9(7):1026–33. doi: 10.1038/s41477-023-01439-4 (PMC10356616; doi:10.1038/s41477-023-01439-4)
Supplement: Supplementary file 1 — Supplementary Figs. 1–4. [file 41477_2023_1439_MOESM1_ESM.pdf]

nature plants

Brief Communication

https://doi.org/10.1038/s41477-023-01439-4

Multiplexed single-cell 3D spatial gene expression analysis in plant tissue using PHYTOMap

In the format provided by the authors and unedited

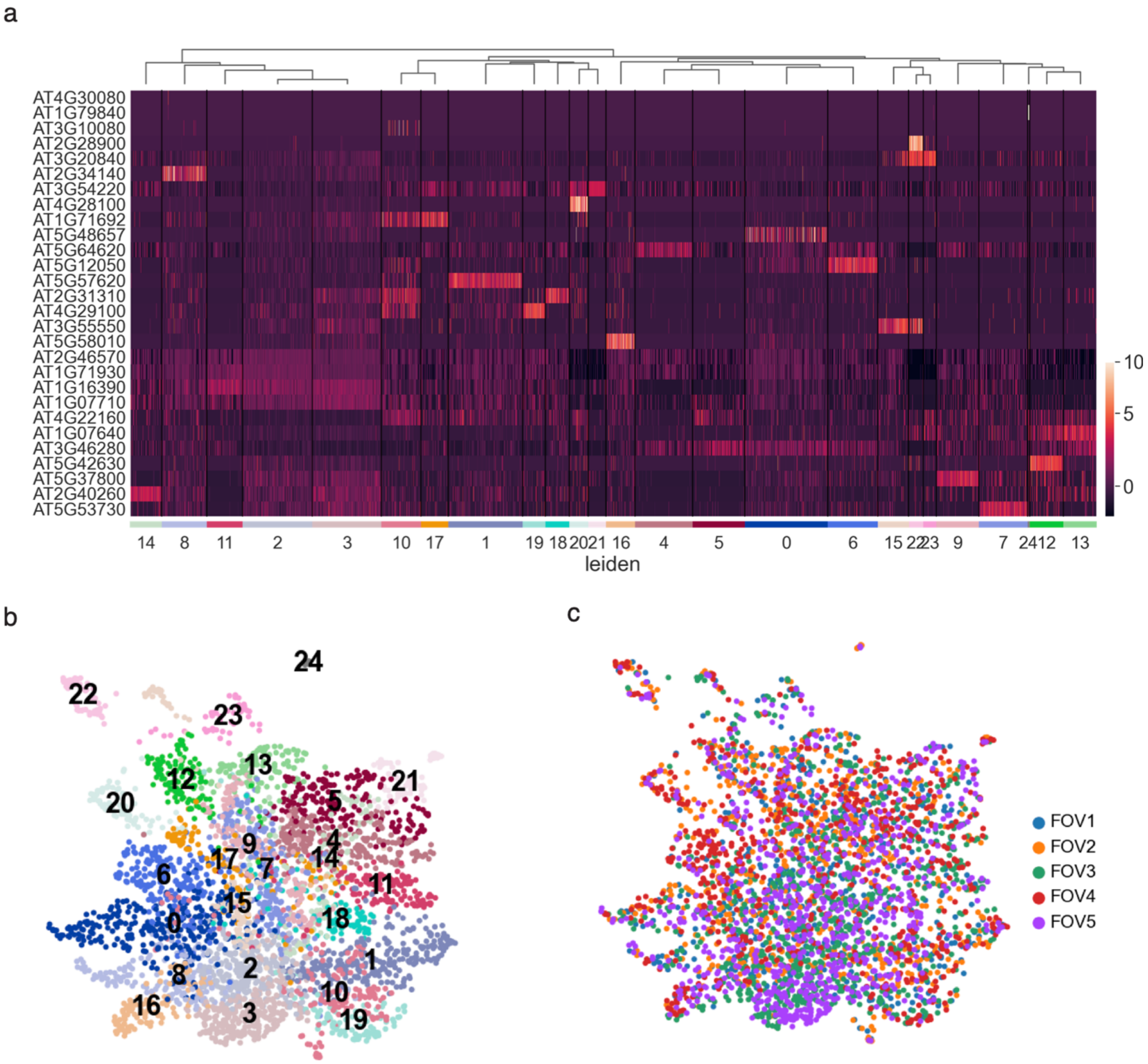

Supplementary Figure 1 | Spatial expression analysis of 28 genes with PHYTOMap

a, Hierarchical clustering of 3608 cells from five root tips based on the relative expression (RE) of 28 genes. Leiden cluster IDs are indicated at the bottom. (b-c), UMAP visualization of the data shown in (a) colored by (b) Leiden clusters and (c) replicates.

a

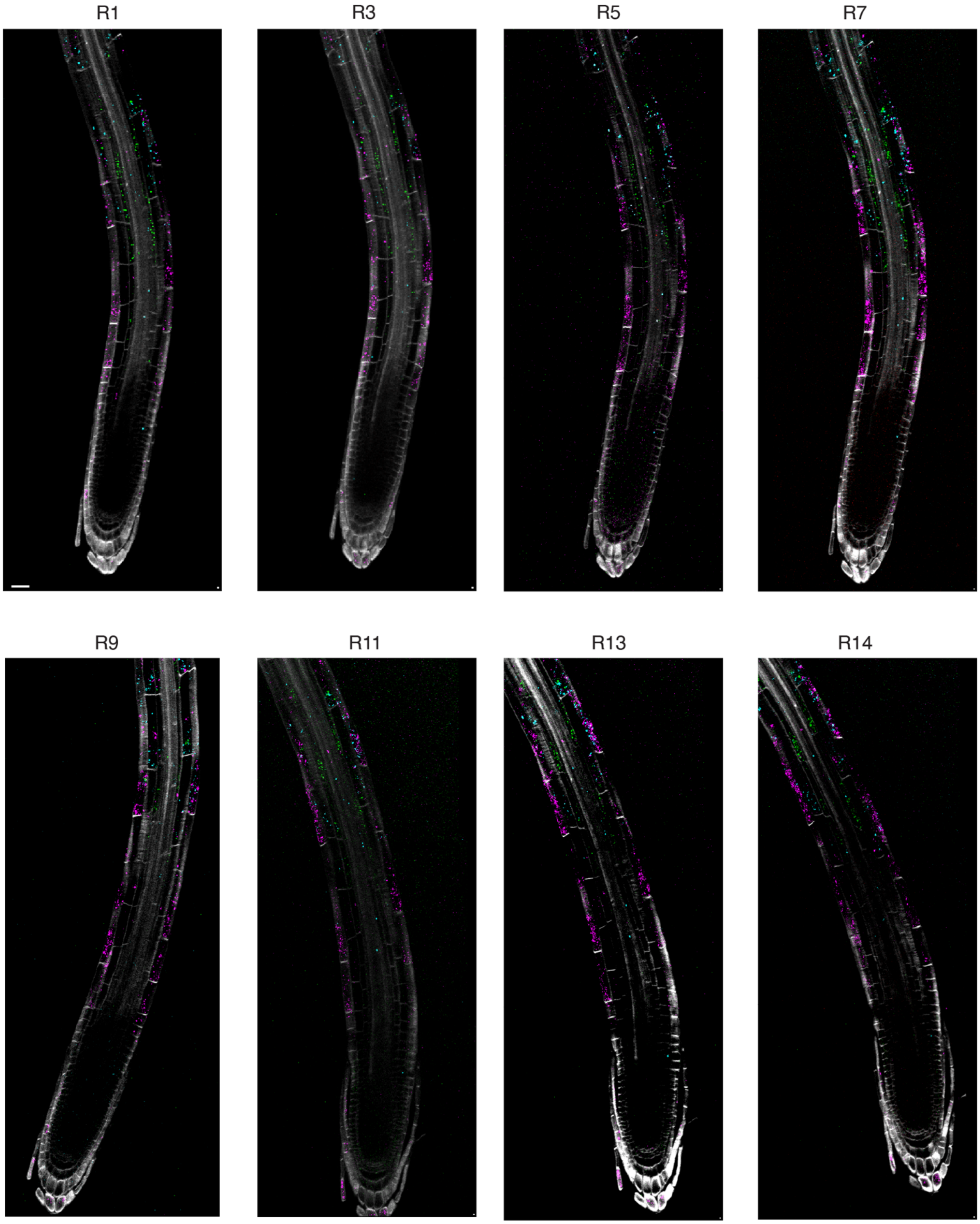

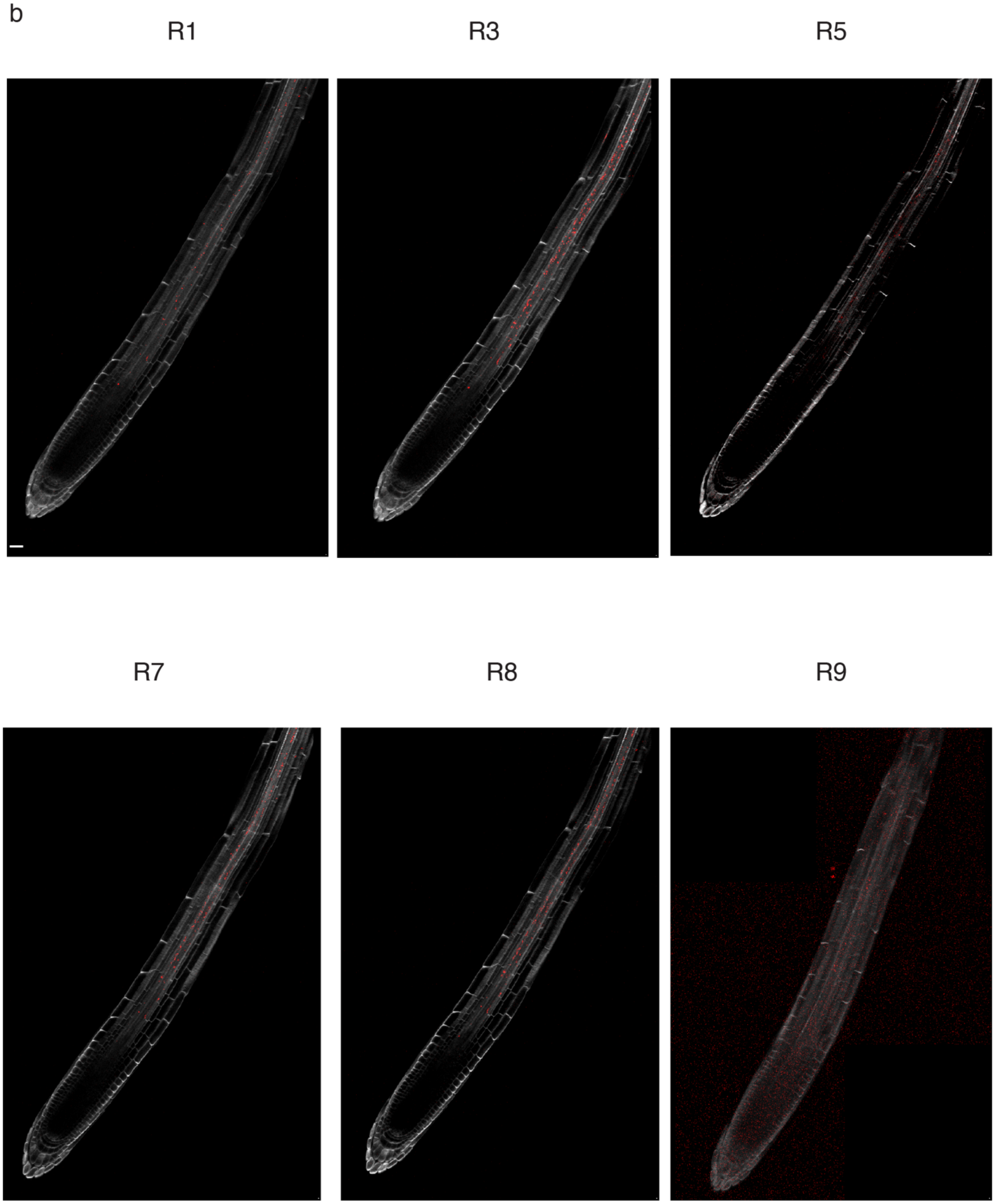

Supplementary Figure 2 | PHYTOMap across 14 rounds of imaging

a, Representative 2D optical sections from each imaging round. Magenta: *AT3G46280* labeled with Alexa Fluor 555. Cyan: *AT2G31310* labeled with Alexa Fluor 647. Green: *AT5G57620* Alexa Fluor 488 b, Red: *AT3G10080* labeled with Alexa Fluor 750. Signal was not detected after the 9th round. Scale bars = 25 μm.

a

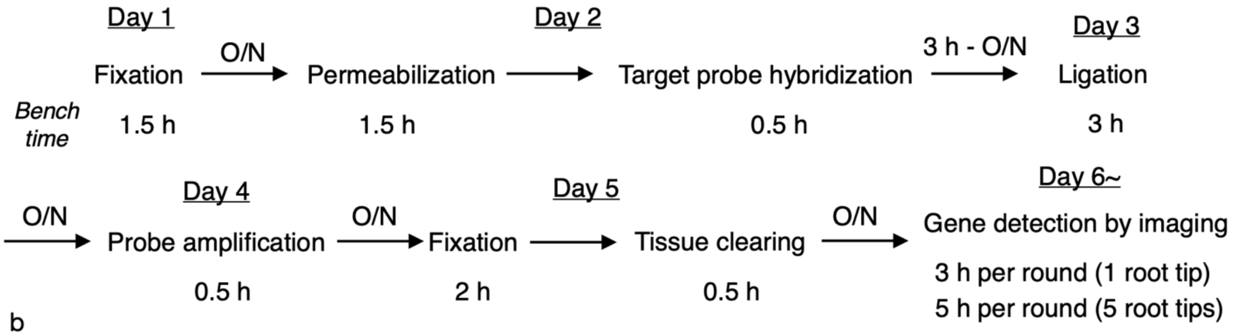

b

Number of genes	Reagent cost	Probe cost	Total cost per experiment	Cost per root tip*
28	$30	$50	$80	$16
96	$30	$200	$230	$46

Supplementary Figure 3 | Timing and cost estimates of PHYTOMap
a, Diagram showing the key steps and timings of PHYTOMap. b, Cost estimates for PHYTOMap experiments at different scales. A sample that fits within a 14 mm diameter circle can be analyzed in one experiment using the protocols presented in this study. *Calculated for five root tips per experiment. It is possible to accommodate more root tips in one experiment.

a

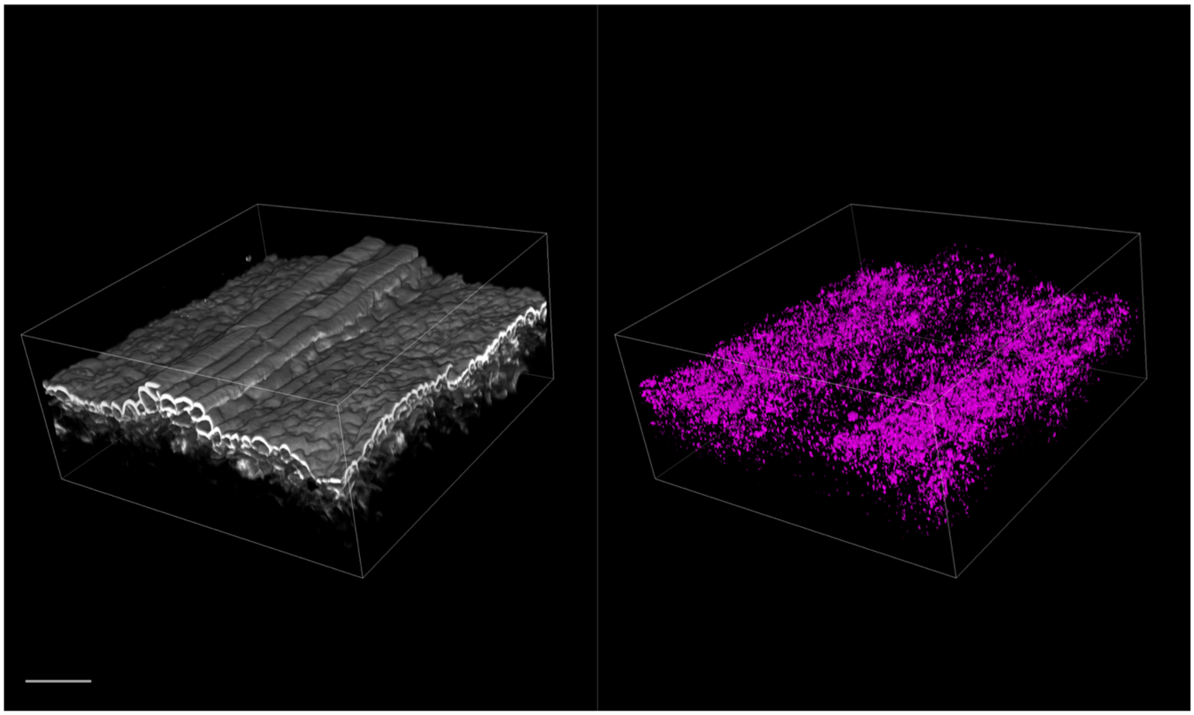

b

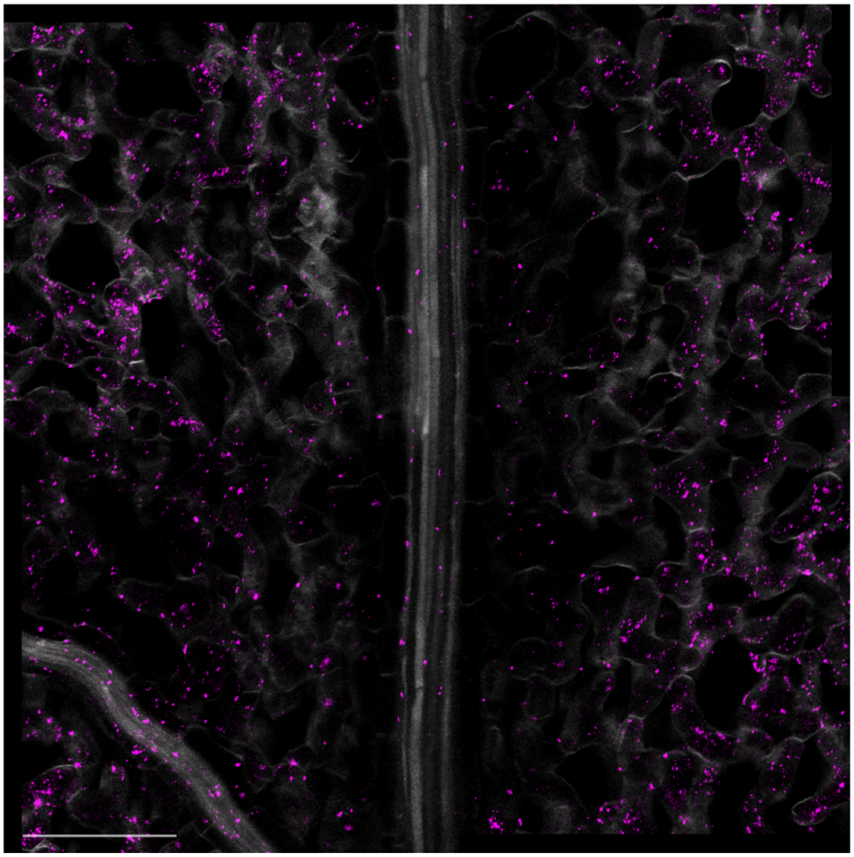

Supplementary Figure 4 | PHYTOMap in Arabidopsis leaves
a, 3D rendering of cell wall staining (leaf) and *UBQ10* (right) images in a whole-mount leaf of Arabidopsis. b, 2D PHYTOMap image of cell wall staining (white) and *UBQ10* (magenta). Scale bars = 100 µm.
